# A novel method for detecting nine hotspot mutations of deafness genes in one tube

**DOI:** 10.1038/s41598-023-50928-1

**Published:** 2024-01-03

**Authors:** Yang Yu, Jun Zhang, Yuxia Zhan, Guanghua Luo

**Affiliations:** Comprehensive Laboratory, The Third Affiliated Hospital of Soochow University, Changzhou, 213003 People’s Republic of China

**Keywords:** Mutation, Molecular medicine

## Abstract

Deafness is a common sensory disorder. In China, approximately 70% of hereditary deafness originates from four common deafness-causing genes: *GJB2*, *SLC26A4*, *GJB3*, and *MT-RNR1*. A single-tube rapid detection method based on 2D-PCR technology was established for nine mutation sites in the aforementioned genes, and Sanger sequencing was used to verify its reliability and accuracy. The frequency of hotspot mutations in deafness genes was analysed in 116 deaf students. 2D-PCR identified 27 genotypes of nine loci according to the melting curve of the FAM, HEX, and Alexa568 fluorescence channels. Of the 116 deaf patients, 12.9% (15/116) carried *SLC26A4* mutations, including c.919-2A > G and c.2168A > G (allele frequencies, 7.3% and 2.2%, respectively). The positivity rate (29.3%; 34/116) was highest for *GJB2* (allele frequency, 15.9% for c.235delC, 6.0% for c.299_300delAT, and 2.6% for c.176-191del16). Sanger sequencing confirmed the consistency of results between the detection methods based on 2D-PCR and DNA sequencing. Common pathogenic mutations in patients with non-syndromic deafness in Changzhou were concentrated in *GJB2* (c.235delC, c.299_300delAT, and c.176-191del16) and *SLC26A4* (c.919-2A > G and c.2168 A > G). 2D-PCR is an effective method for accurately and rapidly identifying deafness-related genotypes using a single-tube reaction, and is superior to DNA sequencing, which has a high cost and long cycle.

## Introduction

Currently, > 1.5 billion people worldwide suffer from hearing loss, including 34 million children, 60% of whom have preventable causes^1^. Congenital hearing loss is a great concern in the medical field. It is one of the most common diseases and is diagnosed in 1–2 newborns per 1,000 live births^2^. Among prepubertal individuals, the incidence increases to 3.5 per 1,000 individuals^3^. This disease is highly heterogeneous. Each person has unique etiological characteristics of hearing loss depending on ethnic, geographic, social, and medical factors. In industrialised countries, genetic factors account for two-thirds of the cases of congenital hearing loss^4^. Most affected individuals (approximately 70%) have non-syndromic hearing loss (NSHL)^5^, which is characterised by no disease other than hearing loss. Autosomal inheritance is the most common mode of inheritance in patients with NSHL^6^. In the Chinese population, it has been demonstrated that NSHL is closely associated with genetic polymorphisms of gap junction protein β2 (*GJB2*), gap junction protein β3 (*GJB3*), solute carrier family 26 member 4 (*SLC26A4*), and mitochondrial DNA (mtDNA) 12S ribosomal RNA (12S rRNA)^7^. Currently, traditional physical screening for hearing defects is widely used, and is associated with the risk of missing detection. The detection of susceptibility genes for deafness can facilitate early detection, intervention, and treatment to clarify the cause, avoid the induction of deafness, and guide the use of drugs. Therefore, detection of deafness susceptibility genes is of great significance. At present, the common screening methods for deafness susceptibility genes are the melting curve^8^, gene chip (including the microarray method)^9^, Sanger sequencing^10^, and second-generation sequencing methods (including whole-exome sequencing, WES)^11^.

Two-dimensional PCR (2D-PCR) is based on the base-quenching probe technology previously developed by our group and is mainly used for the detection of single-nucleotide polymorphisms^12–17^. Presently, our team has successfully established a single-tube method for the differential diagnosis of nine types of high-risk human papillomaviruses using 2D-PCR^18^. In the present study, 2D-PCR was used to detect the *GJB2*, *SLC26A4*, *GJB3*, and *MT-RNR1* genes in 116 deaf patients and to explore the diagnosis rate and mutation patterns of four common pathogenic genes for deafness in Changzhou, Jiangsu, China.

## Materials and methods

### Patients and DNA samples

Whole-blood samples (2.5 ml) from 116 unrelated deaf students in Changzhou Deaf School were collected in June 2010, and stored at -80 ℃ (Changzhou, Jiangsu, China), including samples from 61 male and 55 female patients with an average age of 14.6 ± 4.03 (mean ± SD) years. Each participant provided written informed consent, and informed consent was obtained from the guardians of minors. This study was approved by the Ethics Committee of Changzhou First People’s Hospital (Changzhou, China). All methods were performed in accordance with the relevant guidelines and regulations. Genomic DNA was extracted from peripheral blood samples using a TIANamp Blood DNA kit (Tiangen Biotech Co., Ltd., Beijing, China) according to the manufacturer’s instructions.

### Primers, probes and reaction conditions

The primer design combined the design principles of 2D-PCR^18^ and Amplification Refractory Mutation System PCR (ARMS-PCR). The last base of the 3ʹ end of the primer, with a tag, specifically recognised the wild-type and mutant templates. To improve the amplification specificity, an artificial mismatch base was introduced at the first 3–6 bases of the 3ʹ end of the primer with tag. All primers and probes were designed using Primer Premier 5.0 software. According to the principles of 2D-PCR, three different sequences were designed as probes and labelled with carboxyfluorescein (FAM), hexachloro-fluorescein (HEX), and Alexa568, respectively, and the corresponding three sets of sequences homologous to the corresponding probe sequence were designed as tags. Forward primers were distributed in the three fluorescence channels after being connected to the tags. All primers, tags, and probe sequences are listed in Table 1. Sanger sequencing of all samples and the synthesis of primers and probes were completed by Sangon Biotech Co., Ltd., Shanghai, China; sequencing primers are shown in Table 2. The 2D-PCR reaction mixture contained 2.5 μl 10 × ImmoBuffer (Bioline; Meridian Bioscience, Inc., Cincinnati, OH, USA), 0.75 μl 50 mM MgCl_2_ (Bioline; Meridian Bioscience, Inc.), 0.5 μl 5 U/μl IMMOLASE DNA polymerase (Bioline; Meridian Bioscience, Inc.), 0.7 μl 2.5 mM deoxynucleotide triphosphates (Takara Bio, Inc., Otsu, Japan), 1 μl of 10 μM probe (FAM), 0.8 μl of 10 μM probe (HEX and Alexa568), 0.05–0.2 μl of each 10 μM primer with tag, 0.8–1 μl of the 10 μM primer (see Supplementary Table 1 for details), deionised water to a total volume of 23 μl and, finally, 2 μl of the sample. A reaction mix system was prepared before amplification, and all reagents were stored independently. Because each probe was labelled with a fluorophore, it was stored away from light. Taq enzymes must also be stored independently. The PCR programs for the hot-start reaction system were as follows: pre-incubation at 95˚C for 10 min; 5 cycles at 95˚C for 10 s and 60˚C for 10 s; 35 cycles at 95˚C for 10 s, 72˚C for 1 s and 60˚C for 10 s. The fluorescence acquisition began with heating at 95˚C for 30 s and 30˚C for 4 min; the temperature was gradually increased from 30 to 70˚C with a ramp rate of 0.06˚C/sec, and the fluorescence signal was acquired continuously. Fluorescence intensity was measured using three detection channels: FAM, HEX, and Alexa568. Amplification and melting curve analyses were performed using a SLAN-96S real-time PCR machine (Hongshi Tech, Shanghai, China).

**Table 1:** 2D-PCR primers, tags and probe.

	Primer	Primer sequence (5′ → 3′)	Probe sequence (5ʹ-3ʹ)
F-FAM	919-2A > G W	ccatctacactcccaaactgatcttttcttccttatctcAAATGGCAGTAGCAATTATCG***C***CT	FAM-ccatctacactcccaaactaatcttttcttccttatctc-P
	235delC W	ccatctacactcccaaacgcttcttttcttccttatctcACACGAAGATCAGCTGCA***C***GG	
	176-191del16 W	ccatctacactcccaaagctgcattttcttccttatctcGGAAGTAGTGATCGTAGCACACGTT	
	299_300delAT W	ccatctacactcccagtgaggatgattcttccttatctcCCTTGATGAACTTCCTCTTCTT***A***TCATG	
	1229C > T W	ccatctacactcctgtgagctaagacgcttccttatctcAGTGCTCTCCTGGACGG***A***CG	
	2168A > G W	ccatcttaggatccaaactaatcttttacggacaatctcAGGACACATTCTTTTTGACGGT***A***CA	
	538C > T W	ccatcttgcactagaaactaatctttatgcgaacatctcATCGTGGACTGCTACAT***G***GCCC	
F-HEX	919-2A > G M	cctaatcatcaaccacacgccatcacttcacctatccatTGAAATGGCAGTAGCAATTAT***A***GTCC	HEX-cctaatcatcaaccacttaccatcacttcacctatccat-P
	235delC M	cctaatcatcaaccacgacagatcacttcacctatccatGACACGAAGATCAGCTG***A***AGGC	
	176-191del16M	cctaatcatcaaccaccagatcgtacttcacctatccatGGGGAAGTAGTGATCGTA***C***CTGG	
	299_300delAT M	cctaatcatcaaccacaattttaagattcacctatccatCCTTGATGAACTTCCTCTTCT***A***CTCG	
	1494C > T W	cctaatcatcgttcacttaccatatagctcgacgtccatAATGTCCTTTGAAGTATACTTGAG***C***AGG	
	1555A > G W	cctaatcatcacgtacttaccgcgcgaagttaattccatCCAGTACACTTACCATGTTACGA***A***TTGT	
F-Alexa568	1229C > T M	cacctatccttctatcatttctttccattcaatactcctAGTGCTCTCCTGGA***A***GGCCA	Alexa568-cacctatccttctatcattcctttccattcaatactcct-P
	2168A > G M	cacctatccttctatcattgatttccattcaatactcctGACACATTCTTTTTGACGG***G***CCG	
	1494C > T M	cacctatccttctaaatccaaaccatattcaatactcctAAATGTCCTTTGAAGTATACTTGA***C***GAGA	
	1555A > G M	cacctatccaggtatcattcctttgatcgagtcgctcctCAGTACACTTACCATGTTACGAC***G***TGC	
	538C > T M	cacctatatgcatatcattcctggtgtgctcatactcctCATCGTGGACTGCTACATT***C***CCT	
R	919-2A > G R	CCAATGGAGTTTTTAACATCTTTTG	
	235delC R	CATCTCCCACATCCGGCTAT	
	176-191del16 R	CGACTTTGTCTGCAACACCCT	
	299_300delAT R	CAGCGCTCCTAGTGGCCAT	
	1229C > T R	GGATTCTTCTCTTGTTTTGTGGC	
	2168A > G R	CTCTTGAGATTTCACTTGGTTCTGT	
	538C > T R	GCCCACCATGAAGTAGGTGAAG	
	1494C > T R	TGAAGCGCGTACACACCG	
	1555A > G R	ACTAAAACCCCTACGCATTTATATAGAG	

Lowercase letters represent tag sequences (homologous to the corresponding probe sequence). Underlined text represents the differential sequence between each tag. Bold font and italics represent artificial mutation sites of the specific primer. F, forward primer; M, mutant type; R, reverse primer; W, wild-type.

**Table 2:** Sequence primers.

Sequencing primer	Primer sequence (5′ → 3′)
c.919-2A > G F-Seq	TGCCAGCATTGTAATTTTTTTCC
c.919-2A > G R-Seq	GATTGTGTGTGTGTGCGTGTGT
235–176-299 F-Seq	GCGGACCTTCTGGGTTTTGAT
235–176-299 R-Seq	GTGTTGTGTGCATTCGTCTTTTC
c.2168A > G F-Seq	AGCAATGATGCCACTGCACTC
c.2168A > G R-Seq	TACCGTTTCTAAAATGGAACCTTG
c.1229C > T F-Seq	CCTTCCTCTGTTGCCATTCCT
c.1229C > T R-Seq	GGACCACCACGCAGAGTAGG
1494–1555 F-Seq	AATGGTTTGGCTAAGGTTGTCTG
1494–1555 R-Seq	CTTAAGGGTCGAAGGTGGATTT
c.538C > T F-Seq	GTTCCTCTTCCTCTACCTGCTGC
c.538C > T R-Seq	CTCACAGATGGTGAGTACGATGC

235–176-299 F-Seq was the forward primer for c.235delC, c.176-191del16 and c.299_300delAT. 235–176-299 R-Seq was used as the reverse primer for c.235delC, c.176-191del16, and c.299_300delAT. 1494–1555 F-Seq is the forward primers were m.1494C > T and m.1555A > G. 1494–1555 R-Seq were the reverse primers m.1494C > T and m.1555A > G. F, forward primer; R, reverse primer.

### Statistical analysis

Results are expressed as mean ± the SD. Chi-square test was used to compare the detection rates of hereditary deafness gene mutations between Changzhou and other areas in China. GraphPad Prism 8.0 software (GraphPad Software; DotMatics, Boston, MA, USA) was used to perform statistical analyses. P < 0.05 was considered statistically significant.

### Ethics approval

Each participant provided written informed consent, and informed consent was obtained from their guardians. This study was approved by the Ethics Committee of Changzhou First People’s Hospital (Changzhou, China).

## Results

### 2D-PCR melting temperatures

**Figure 1:**
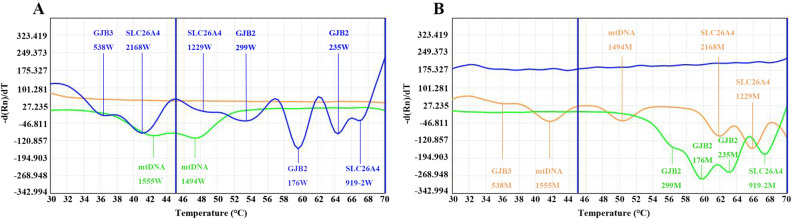
2D-PCR plasmid amplification melting curves. (**A**) Melting curve of wild-type mixed plasmid (1 × 10^5^ copies). (**B**) Melting curve of mutant mixed plasmid (1 × 10^5^ copies). 919–2, c.919-2A > G; 235, c.235delC; 176, c.176-191del16; 299, c.299_300delAT; 1229, c.1229C > T; 2168, c.2168A > G; 538, c.538C > T; 1494, m.1494C > T; 1555, m.1555A > G; 2D-PCR, two-dimensional PCR; M, mutant type; W, wild-type. Blue melting curve represents the FAM channel; green melting curve represents the HEX channel; orange melting curve represents the Alexa568 channel.

The genotypes of nine loci in 116 deaf patients were detected using 2D-PCR and Sanger sequencing. Wild-type or homozygous mutant plasmid (5 μl each; 1 × 10^6^ copies) from the nine sites were mixed to a final concentration of approximately 1 × 10^5^ copies, and the mixed plasmids were used as templates for 2D-PCR amplification. The melting curves are shown in Fig. 1A,B. The melting temperatures of different genotypes at each locus detected by 2D-PCR are listed in Table 3. According to the melting curve and the corresponding melting temperature, the melting curves of FAM channels were all wild-type; those of the HEX channel at < 48˚C were wild-type and that at > 48˚C was mutant type; and the melting curves of the Alexa568 channel were all mutant types.

**Table 3:** The melting temperatures of different genotypes at each locus detected by 2D-PCR technology.

Mutation site	Wild-type		Mutant-type	
	Tm values (℃)	Channel	Tm values (℃)	Channel
c.919-2A > G	66.8	FAM	67.2	HEX
c.235delC	64.4	FAM	63.2	HEX
c.176-191del l6	59.6	FAM	59.6	HEX
c.299_300delAT	53.2	FAM	56.4	HEX
c.1229 C > T	48.4	FAM	66	Alexa 568
c.2168A > G	41.2	FAM	62	Alexa 568
c.538C > T	36.4	FAM	50.4	Alexa 568
m.1494C > T	47.2	HEX	41.6	Alexa 568
m.1555A > G	42.4	HEX	35.6	Alexa 568

### Frequency of nine hotspot mutations

A total of 50 of 116 patients (43.1%) carried at least one genetic deafness-associated variant (one patient had mutations in both GJB3 and MT-RNR1). Table 4 shows the genotypes of 116 deaf patients from the Changzhou area. Table 5 shows the allele frequencies of the nine hotspot mutations associated with deafness.

**Table 4:** Genotypes of 116 deafness patients from Changzhou area.

Gene	Genotype	n (%)
*GJB2*, total		34 (29.3)
Homozygous, total		13 (11.2)
	c.235delC/c.235delC	10
	c.176-191del16/ c.176-191del16	1
	c.299_300delAT/ c.299_300delAT	2
Compound heterozygous, total		10 (8.6)
	c.235delC/ c.299_300delAT	6
	c.235delC/ c.176-191del16	3
	c.176-191del16/ c.299_300delAT	1
Heterozygous, total		11 (9.5)
	c.235delC/wt	8
	c.299_300delAT/wt	3
*SLC26A4*, total		15 (12.9)
Homozygous, total		3 (2.6)
	c.919–2 A > G / c.919–2 A > G	3
Compound heterozygous, total		4 (3.4)
	c.919-2A > G/ c.2168A > G	4
Heterozygous, total		8 (6.9)
	c.919-2A > G/wt	7
	c.2168A > G/wt	1
*MT-RNR1*, total		2 (1.7)
Homoplasmic, total		2 (1.7)
	m.1555A > G	2

**Table 5:** The allele frequencies of nine hotspot mutations of deafness.

Gene name	Mutation site	Type (n = 116)		Allele frequency (%)
		Heterozygous*	Homozygotes	
*GJB2*	c.235delC	17	10	15.9
	c.176-191dell6	4	1	2.6
	c.299_300delAT	10	2	6.0
*SLC26A4*	c.919-2A > G	11	3	7.3
	c.2168A > G	5	0	2.2
	c.1229C > T	0	0	0
*GJB3*	c.538C > T	0	0	0
*MT-RNR1*	m.1494C > T	0	0	0
	m.1555A > G	0	2	1.7

*Heterozygotes for different genes were counted separately.

### GJB2

**Figure 2:**
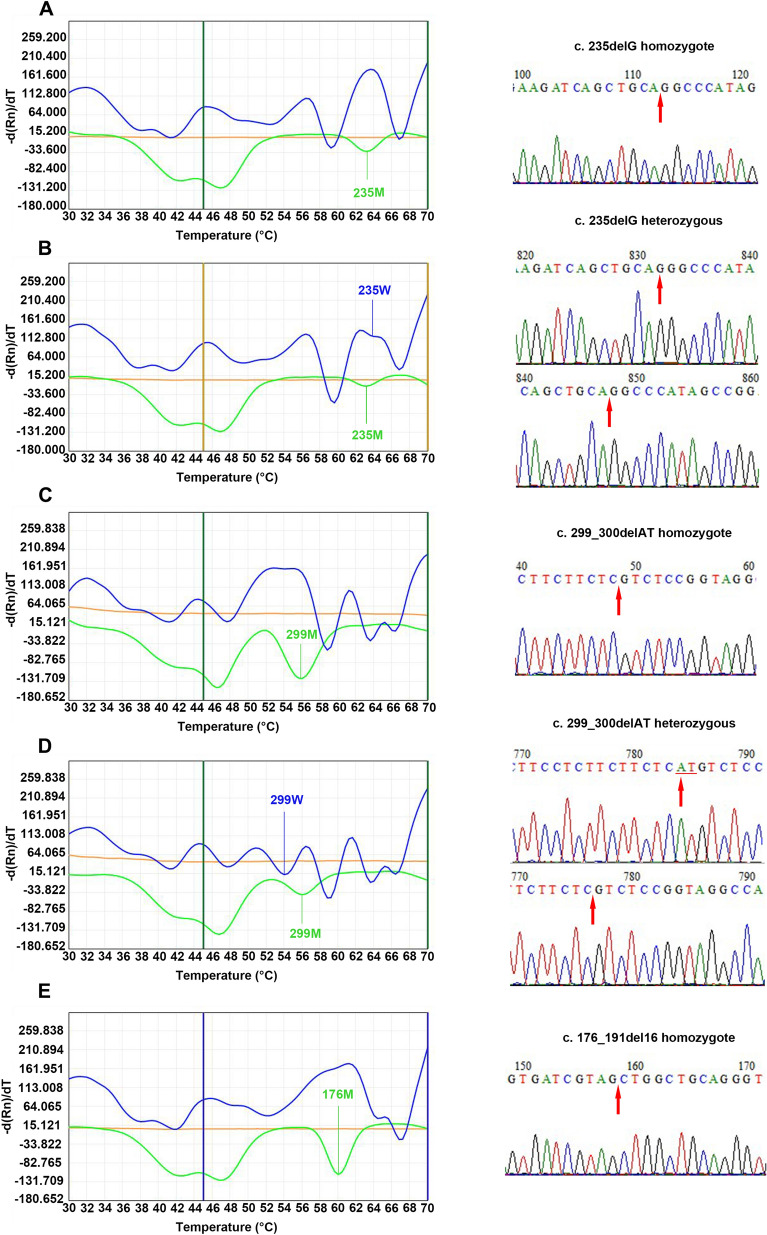
Melting curve of *GJB2* gene single point mutation and its corresponding Sanger sequencing map. (**A**) c.235delC/c.235delC homozygous mutation. (**B**) c.235delC/wt heterozygous mutation. (**C**) c.299_300delAT/c.299_300delAT homozygous mutation. (**D**) c.299_300delAT/wt heterozygous mutation. (**E**) c.176-191del16/c.176-191del16 homozygous mutation. The mutation site is underlined and the red arrow points to the mutation position. 235, c.235delC; 176, c.176-191del16; 299, c.299_300delAT; *GJB2*, gap junction protein β2; M, mutant type; W, wild-type.

**Figure 3:**
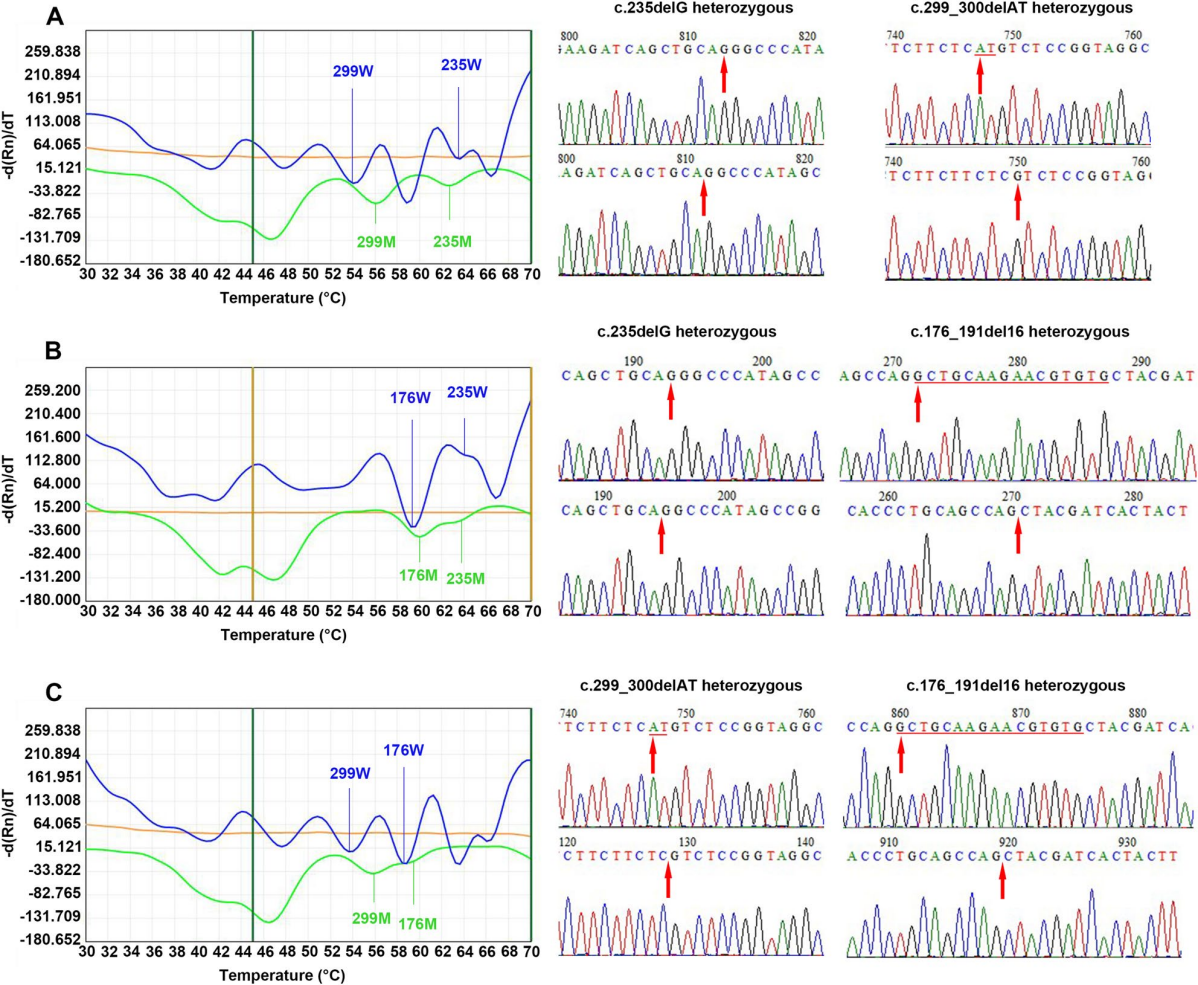
Melting curves and corresponding Sanger sequencing results for the *GJB2* compound heterozygous mutation. (**A**) c.235delC/c.299_300delAT. (**B**) c.235delC/c.176-191del16. (**C**) c.176-191del16/c.299_300delAT. The mutation site is underlined and the red arrow points to the mutation position. 235, c.235delC; 176, c.176-191del16; 299, c.299_300delAT; *GJB2*, gap junction protein β2; M, mutant type; W, wild-type.

**Figure 4:**
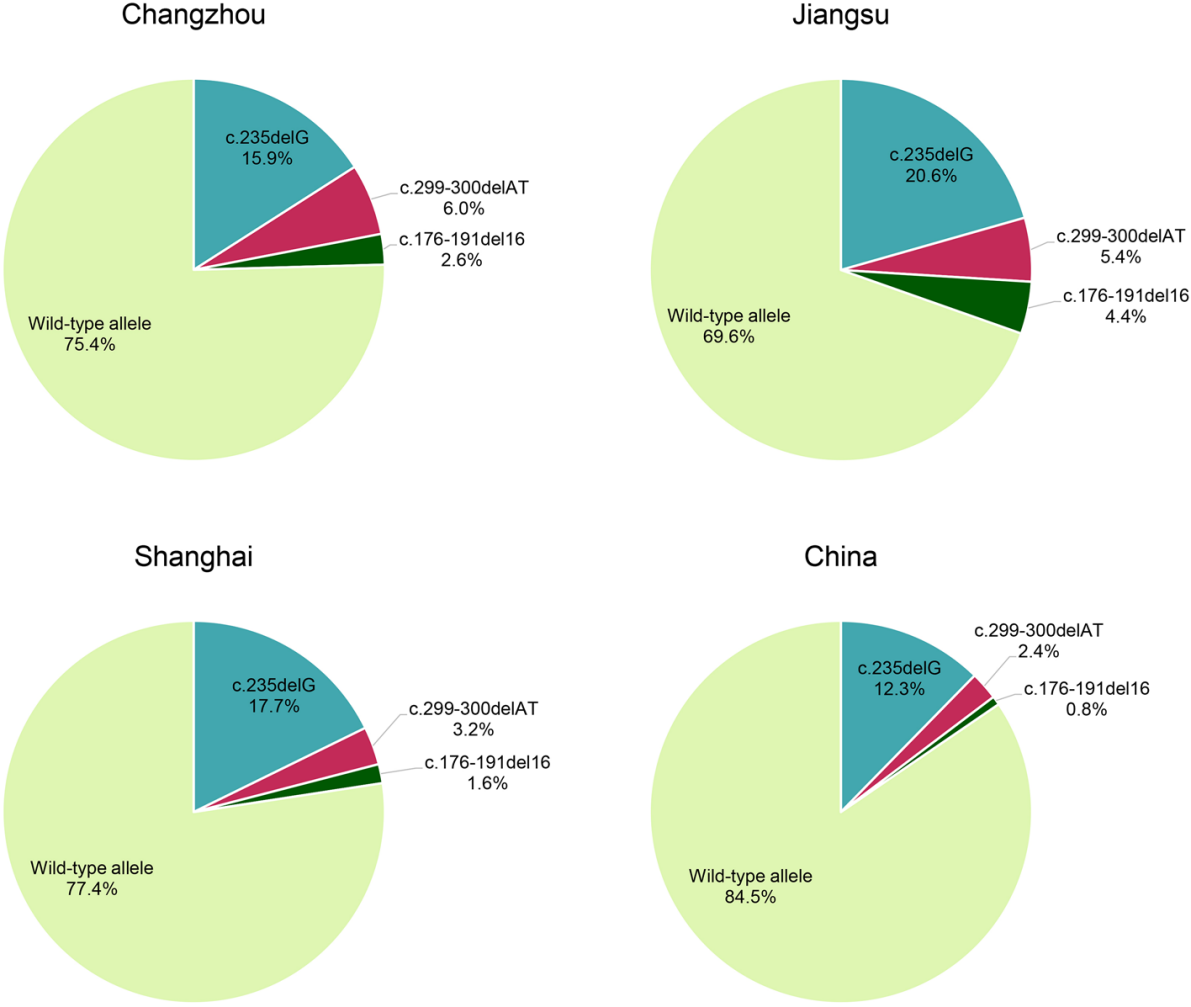
Distribution of allele frequencies of three hotspot mutations (c.235delC, c.299_300delAT and c.176-191del16) of the *GJB2* gene in the study cohort from Changzhou and its surrounding provinces and cities, as well as in China (15,16). The participants in all regions were deaf patients. *GJB2*, gap junction protein β2.

A total of 34 patients with *GJB2* gene mutations were detected by the 2D-PCR and Sanger sequencing, including 13 homozygous and 21 heterozygous mutations. There were 10 homozygous (the results of 2D-PCR and sequencing are shown in Fig. 2A) and 17 heterozygous (Fig. 2B) c.235delC mutations (positivity rate, 23.3%; frequency of mutant alleles, 15.9%), 2 homozygous (Fig. 2C) and 10 heterozygous (Fig. 2D) mutations of c.299_300delAT (positivity rate, 10.3%; frequency of mutant alleles, 6.0%), and 1 homozygous (Fig. 2E) and 4 heterozygous mutations (all double-site heterozygous mutations) of c.176-191del16 (positivity rate, 4.3%; frequency of mutant alleles, 2.6%). A patient with c.35insG was identified using Sanger sequencing; this patient also carried the c.299_300delAT mutation (see Supplementary Fig. 1 for details). In addition, 10 patients were compound heterozygous, accounting for 29.4% (10/34) of the total *GJB2* gene mutations. Of these, 17.6% (6/34) carried c.235delC + c.299_300delAT (Fig. 3A), 8.8% (3/34) carried c.235delC + c.176-191del16 (Fig. 3B), and 2.9% (1/34) carried c.299_300delAT + c.176-191del16 (Fig. 3C).

The positivity rate and allele frequency of c.235delC were the highest (23.3% and 15.9%, respectively), similar to the results of studies performed in Jiangsu (20.6%; χ^2^, 1.58; P > 0.05) and Shanghai (17.7%; χ^2^, 0.12; P > 0.05)^19^. However, the positivity rate was significantly higher than that reported for 3,004 patients from 26 regions in China (16.3%; χ^2^, 4.01; *P* < 0.05), but there was no significant difference in allele frequencies (15.9 vs. 12.0%; χ^2^, 3.26; *P* > 0.05)^20^.

In the present study, the positivity rates of c.299_300delAT and c.176-191del16 in Changzhou were 10.3 and 4.3%, respectively, and the allele frequencies were 6.0 and 2.6%, respectively, which were not significantly different from those reported in Jiangsu and Shanghai. However, the positivity rates and allele frequencies of c.299_300delAT and c.176-191del16 in this study were significantly higher than those of 2,063 patients from 23 provinces in China (c.299_300delAT: positivity rate, 4.4%; χ^2^, 8.81; *P* < 0.01; allele frequency, 2.4%; χ^2^, 11.75; *P* < 0.001; c.176-191del16: positivity rate, 1.4%; χ^2^, 6.03; *P* < 0.05; allele frequency, 0.75%; χ^2^, 8.79; *P* < 0.01)^19^. Figure 4 shows the gene frequencies of three hotspot mutations in the *GJB2* gene in the present cohort from Changzhou and its surrounding areas.

### SLC26A4

**Figure 5:**
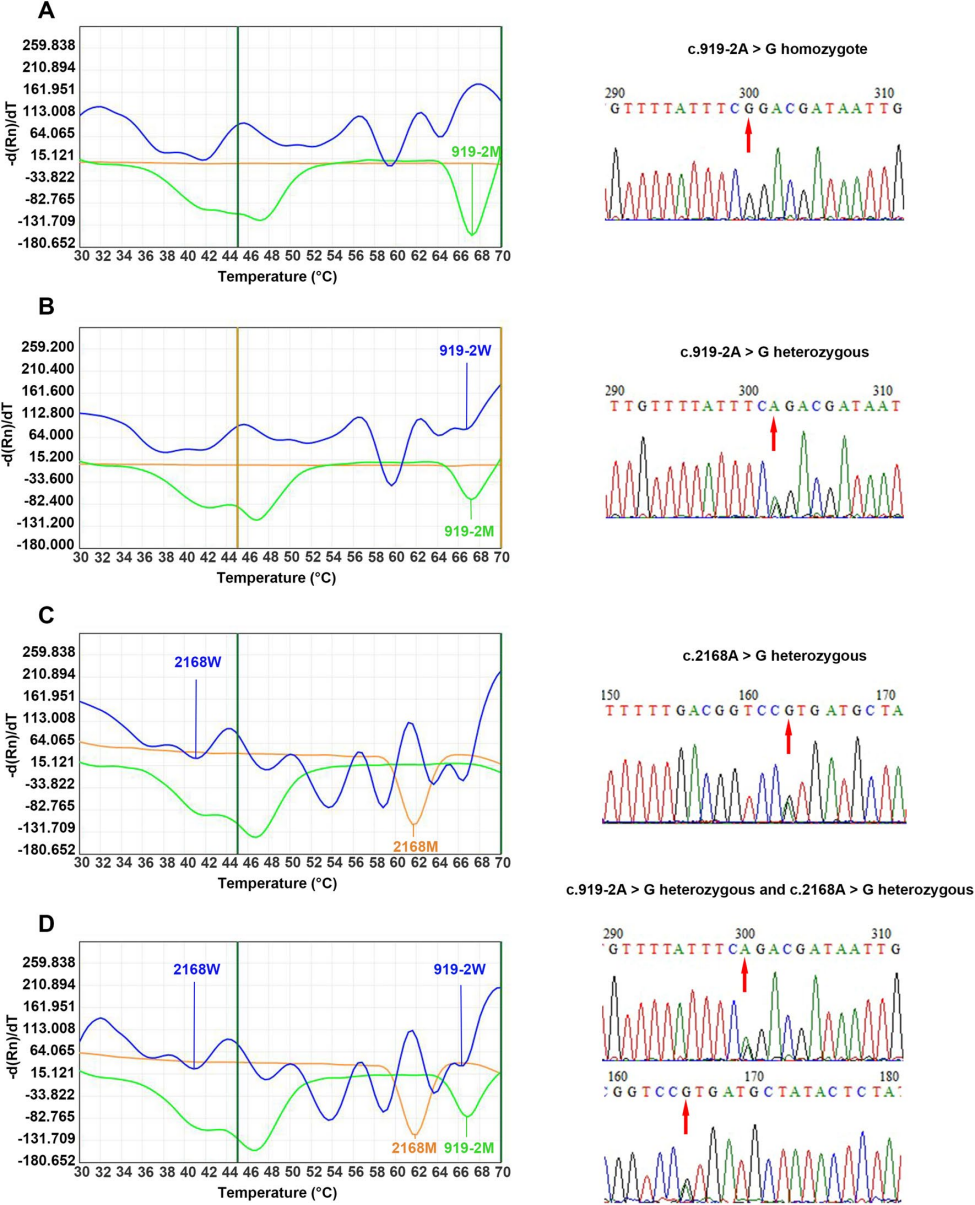
Melting curves of *SLC26A4* genotypes and their corresponding Sanger sequencing results. (**A**) c.919-2A > G/c.IVS7-2A > G homozygous mutation. (**B**) c.919-2A > G/wt heterozygous mutation. (**C**) c.2168A > G/wt heterozygous mutation. (**D**) c.919-2A > G/c.2168A > G compound heterozygous mutation. The red arrow points to the mutation site. 919–2, c.919-2A > G; 2168, c.2168A > G; M, mutant type; *SLC26A4*, solute carrier family 26 member 4; W, wild-type.

**Figure 6:**
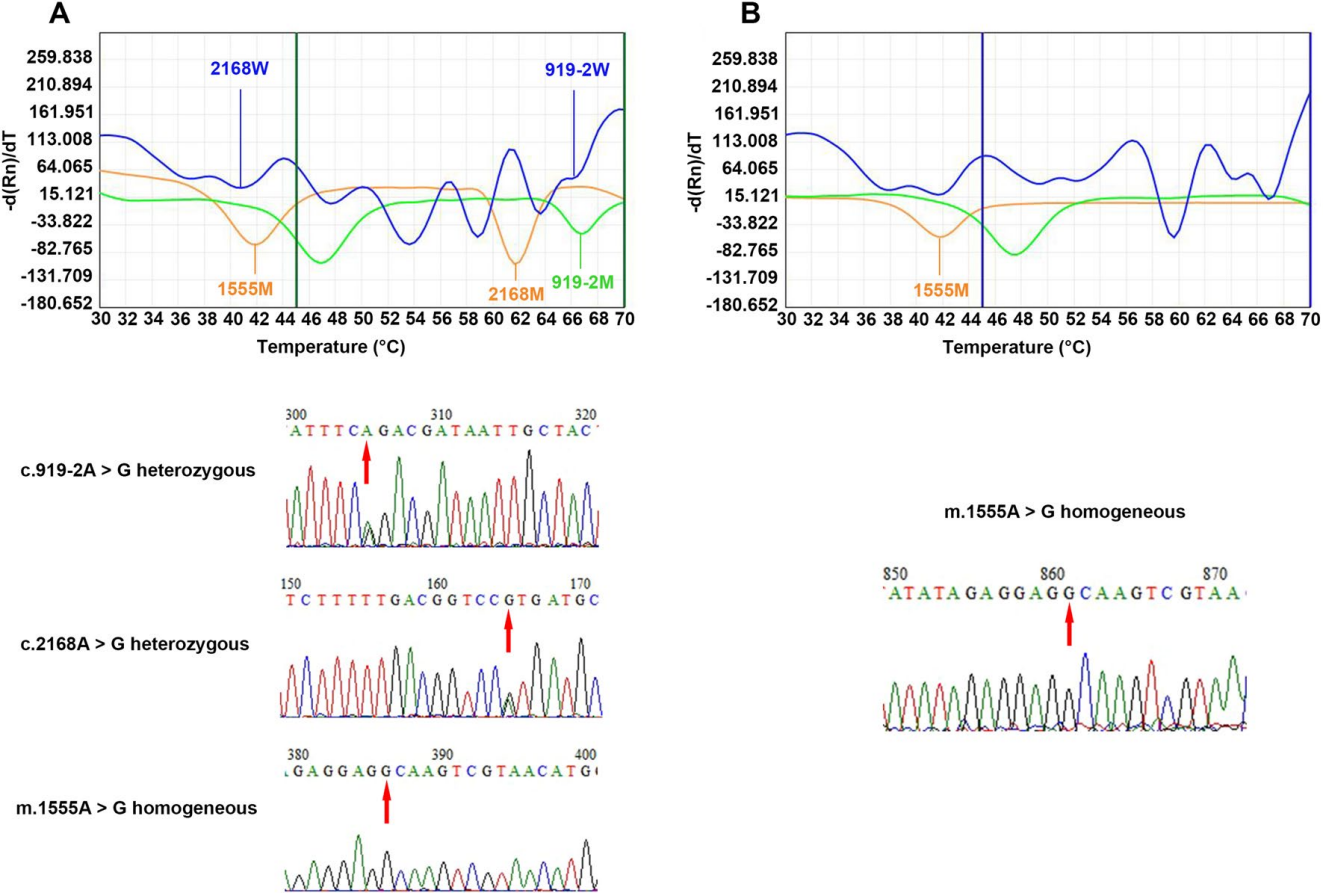
Melting curves and sequencing maps of patients with three mutation sites and the m.1555A > G homogenous variant alone. (**A**) *SLC26A4* c.919-2A > G + c.2168A > G + *MT-RNR1* gene m.1555A > G. (**B**) m.1555A > G homoplasmic. The red arrow points to the mutation site. 919–2, c.919-2A > G; 2168, c.2168A > G; 1555, m.1555A > G; M, mutant type; *mtDNA*, mitochondrial DNA; *SLC26A4*, solute carrier family 26 member 4; W, wild-type.

*SLC26A4* mutations were detected in 15 of the 116 patients. There were 3 homozygous (Fig. 5A) and 11 heterozygous (Fig. 5B) c.919-2A > G mutations (positivity rate, 12.1%; frequency of mutant alleles, 7.3%), and 5 heterozygous (Fig. 5C) c.2168A > G mutations (positivity rate, 4.3%; frequency of mutant alleles, 2.2%). No mutation at position c.1229C > T was detected. Four patients (26.7%; 4/15) exhibited mutations at multiple sites in *SLC26A4*. Of these patients, three carried the c.919-2A > G + c.2168A > G mutation (Fig. 5D). Another patient had three mutations simultaneously (*SLC26A4* c.919-2A > G + c.2168A > G + *MT-RNR1* gene m.1555A > G; Fig. 6A).

**Figure 7:**
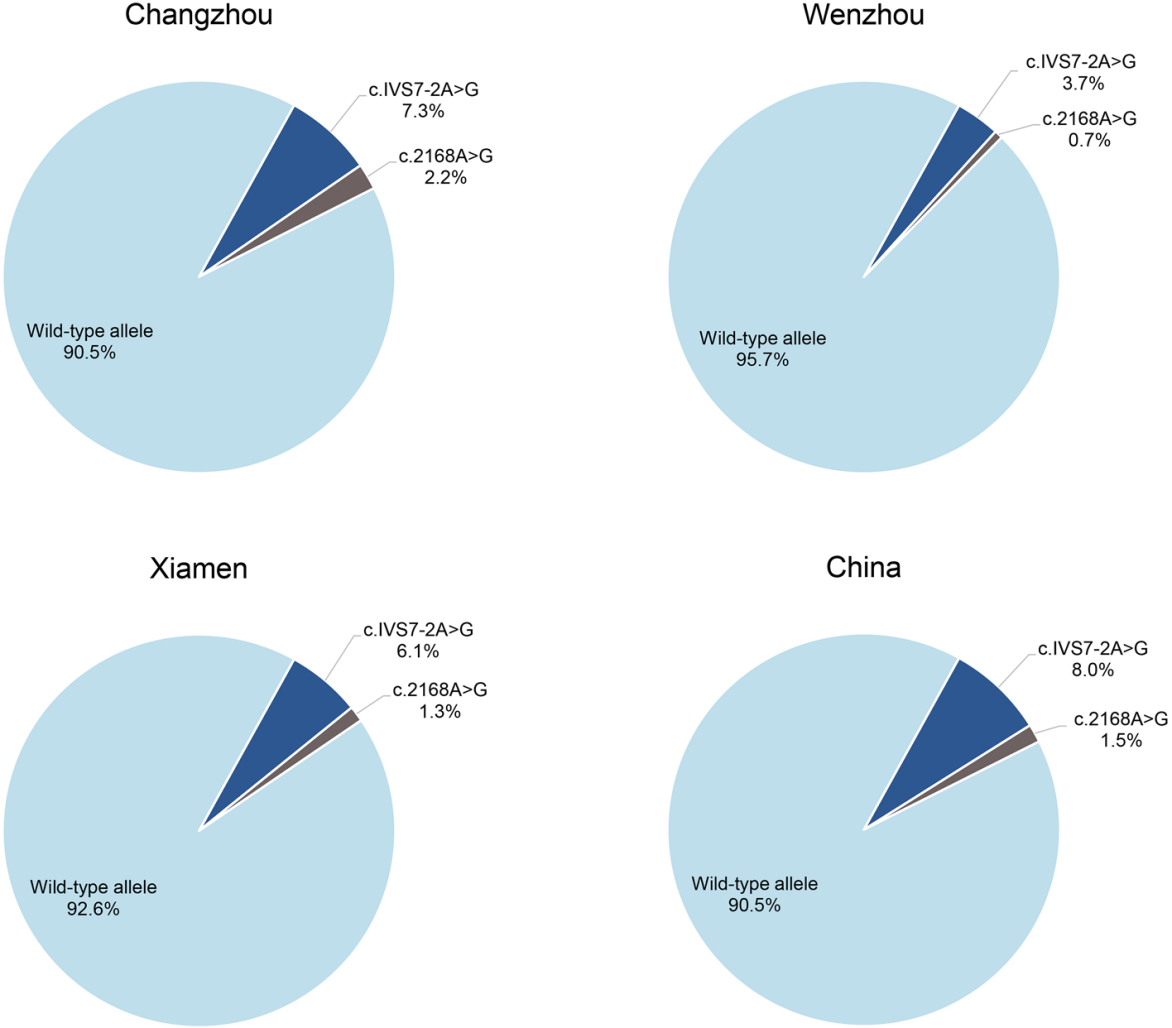
Distribution of allele frequencies of two hotspot mutations (c.919-2A > G and c.2168A > G) of the *SLC26A4* gene in the study cohort from Changzhou and its surrounding provinces and cities, as well as the whole country (15–17). The participants in all regions were deaf patients. *SLC26A4*, solute carrier family 26 member 4.

The present study revealed the highest allele mutation rate of c.919-2A > G (7.3%) in the *SLC26A4* gene, which was significantly higher than that reported for cohorts from other cities in East China, such as Wenzhou^21^ (3.66%; χ^2^, 24.64; P < 0.0001) and Xiamen^22^ (6.13%; χ^2^, 5.19; *P* < 0.05). In 2,352 patients with non-syndromic deafness from 27 cities in China, the positivity rates of three known pathogenic sites of the *SLC26A4* gene, c.919-2A > G, c.2168A > G, and c.1229C > T, were 11.52, 2.5%, and 0.51%, respectively. The allele frequencies were 8.01, 1.51, and 0.25%, respectively^23^. Figure 7 shows the gene frequencies of three hotspot mutations in the *SLC26A4* gene in the present cohort from Changzhou and its surrounding areas. The positivity rate and allele frequency of the two major *SLC26A4* mutation sites, c.919-2A > G and c.2168A > G, in the present cohort from the Changzhou area were similar to those at the national level, and there was no significant difference.

### MT-RNR1 and GJB3

A total of 2 cases of *MT-RNR1* gene mutation were detected (both m.1555A > G homoplasmic mutation; Fig. 6B). The positivity and allelic mutation rates were 1.7% and 1.7%, respectively. The mutation at c.538C > T of the *GJB3* gene was not detected in any patients.

### Comparison of 2D-PCR and Sanger sequencing results

The 2D-PCR method for the 35delG locus of *GJB2* was not included in this study. Therefore, the 2D-PCR results for the nine loci were consistent with the Sanger sequencing results. The kappa test showed complete concordance between 2D-PCR and the Sanger sequencing methods (*k* = 1; *P* = 0.000).

## Discussion

Deafness is a common sensory disorder with a complex aetiology; approximately 60% of deafness is hereditary^24^. According to the Report on Prevention and Treatment of Birth Defects in China in 2012^25^, hearing disabilities accounted for 24.97% of the disabled population in China. With the improvement of healthcare consciousness of eugenics, the causes of deafness caused by environmental factors have been gradually reduced, and the proportion of hereditary deafness is gradually increasing; thus, the detection of deafness gene mutations is increasingly important.

The present study demonstrates that the *GJB2* gene had the highest mutation detection rate. Connexin 26 (Cx26), encoded by *GJB2*, is a gap junction protein composed of six monomers^26^. In fact, deleterious mutations in the gene *GJB2* encoding Cx26 underlie the most common form of non-syndromic congenital deafness, making it an important putative target for gene therapy^27,28^. Research demonstrated that *GJB2* gene mutations can cause potassium repolarization reflux disorders, resulting in hearing loss^29^. If newborn deafness gene detection reveals that the infant exhibits a double mutation in the *GJB2* gene at any site of c.35delG, c.176-191del16, c.235delC, or c.299_300delAT, the auditory nerve, auditory conduction pathway, and speech centre of the infant are normal, and a good rehabilitation effect can be obtained by cochlear implantation^30^. *GJB2* is the most common deafness-causing gene in many ethnic groups. Among *GJB2* mutations, c. 235delC is the most common in Asian populations^31–34^. The positivity rates of *GJB2* c.235delC, c.299_300delAT, and c.176-191del16 have been reported to be 16.3^20^, 4.36, and 1.4%, respectively^19^. The positivity rates of *GJB2* c.235delC, c.299_300delAT, and c.176-191del16 were 23.3, 10.3, and 4.3%, respectively, in patients with non-syndromic hearing loss in the present study cohort from Changzhou. In addition, nine patients had c.235delC with other mutations, accounting for 52.9% (9/17) of the total heterozygous mutations, including six cases of c.235delC + c.299_300delAT and three cases of c.235delC + c.176-191del16. The heterozygous mutation c.235delC is often accompanied by another mutation that causes deafness. Therefore, the *GJB2* c.235delC, c.299_300delAT, and c.176-191del16 loci can be used as the focus of genetic screening for deafness in Changzhou to avoid blind screening.

In addition, Sanger sequencing in the present study revealed a novel compound heterozygous mutation, c.35insG + c.299_300delAT, in a study cohort from the Changzhou area. It has been reported that 35insG insertions lead to frameshift mutations in sporadic deafness cases^35–37^. However, to the best of our knowledge, no compound heterozygous c.35insG + c.299_300delAT mutations have been reported. c.35insG caused the stop codon to advance to position 47, and the translated amino acid was shortened accordingly, resulting in a truncated protein that could not function normally. Because the 3ʹ end of 35delG site has GGGGGG, which increases the difficulty of specific primer design, we will optimise the specific primer sequence of this site. In future experiments, 2D-PCR detection of c.35delG and c.35insG will be performed.

Notably, none of the patients included in the present study had a history of cancer. Previous studies have found that GJB2 is highly expressed in lung adenocarcinoma^38^, cervical cancer^39^, breast cancer^40^, and pancreatic cancer^41^, and is associated with poor prognosis. However, GJB2-related deafness susceptibility genes are not associated with cancer. This may be related to the increased expression of GJB2 and the degree of differentiation of different cancers, indicating that GJB2 can be used as a potential prognostic marker and therapeutic target for the poor survival of cancer patients. However, in deaf patients, GJB2 mutations may cause abnormal expression of GJB2 mRNA or protein, resulting in the loss of GJB2 expression^11^. Therefore, GJB2 mutations associated with deafness may play a role in tumour suppression to some extent.

Mutation of the *SLC26A4* gene is responsible for enlargement of the vestibular aqueduct^42^. In China, approximately 97% of enlarged vestibular aqueducts are caused by *SLC26A4* gene mutations^43^. Among the *SLC26A4* gene mutations, c.919–2 A > G has the highest mutation rate in the Chinese population. In 2,352 patients with non-syndromic deafness from 27 cities in China, the positivity rates of c.919–2 A > G, c.2168 A > G, and c.1229 C > T were 11.5, 2.5, and 0.5%, respectively^23^. In the present study, the positivity rates of these three loci were 7.3, 2.2, and 0%, respectively. Compared with the aforementioned reports, the mutation trend at each site was similar; however, no mutation was found at the c.1229 C > T site in the study cohort from the Changzhou area, which may be related to different genetic backgrounds. Furthermore, four of the five patients (80%) with heterozygous c.2168 A > G mutations also had a heterozygous c.919–2 A > G mutation. Therefore, the c.919–2 A > G and c.2168 A > G loci are hotspot mutations in patients with *SLC26A4* gene mutations in the Changzhou area, which together affect the hearing of patients with large vestibular aqueducts.

mtDNA is the cytoplasmic genome that is independent of nuclear chromosomes. *MT-RNR1* m.1555A > G and m.1494C > T mutations are closely associated with aminoglycoside-induced deafness^44^. Mutant carriers are extremely sensitive to aminoglycosides and tinnitus and severe deafness may occur even with low doses of aminoglycosides. Epidemiological data demonstrate that mtDNA m.1555A > G and m.1494C > T are the two main pathogenic loci of the MT-RNR1 gene in the Chinese deaf population, with carrying rates of 3.43^45^ and 0.41%^46^. In the present study, the positivity rates of m.1555A > G and m.1494C > T were 1.7% and 0%, respectively; the difference from our previous data was due to the loss of a sample from a patient with a homoplasmic A-to-G mutation at position 1555 of the mtDNA in the present study, with the total number of participants changing from 117 to 116. Although this result was lower than the national level, the difference was not statistically significant. This was due to the different numbers of participants and different genetic backgrounds caused by regional and ethnic differences. In the Changzhou non-syndromic deafness population, the m.1555A > G mutation is common in patients with drug-induced deafness. In addition, among the four patients with c.2168 A > G and c.919–2 A > G mutations, one patient with congenital deafness had m.1555 A > G homoplasmic mutations. Because we did not collect blood samples from the deaf patient's parents, we were unable to accurately determine the inheritance pattern. Studies show that human mitochondrial DNA is maternally inherited^47^. Therefore, we speculate that the m. 1555A > G mutation originated from the mother. SLC26A4 gene (7q31) has a recessive inheritance^48^, and pathological mutations of this allele can lead to diseases. Both c.919-2A > G and c.2168A > G are located in SLA26A4. However, this patient had heterozygous mutations at c.919-2A > G and c.2168A > G. Therefore, we speculated that at least one of the parents of this deaf patient carried the G alleles at c.919 and c.2168.

At present, a series of molecular biology methods based on PCR technology are mainly used to detect deafness susceptibility genes, including restriction fragment length polymorphisms, denaturing high-performance liquid chromatography, allele-specific PCR, SNaPshot sequencing, high-throughput sequencing, gene chips, and direct DNA sequencing. WES^11^ can directly sequence the protein-coding sequence and determine the variation that affects the protein structure; however, it requires expensive sequencing equipment and corresponding analysis software. This method is more suitable for screening many pathogenic genes and identifying new mutation sites without clear diagnostic information. Although microarray technology can detect more deafness-related mutation sites at the same time^9^, this method also requires a Microarray Scanner and detection system, which increases the cost of detection and can produce false positives and reduce the accuracy of detection^49^. Sanger sequencing, microarray chips, and WES detection methods also have a common problem: the lid of the reaction tube must be opened to remove the PCR products for follow-up testing, which increases the risk of laboratory contamination. These methods have made great contributions to the discovery and identification of deafness susceptibility genes; however, they have not been widely accepted in clinical practice because they are time-consuming, require expensive equipment and consumables, and cannot detect multiple mutation sites of different genes simultaneously. Deafness susceptibility gene detection can change the traditional passive treatment of deafness diseases into active prevention, which may comprise early detection and intervention and therefore may help prevent deafness. In the present study, 2D-PCR technology was used to detect the genotypes of nine mutation sites in deafness-related genes in a single, closed tube and one-time reaction. This technology amplifies and identifies target genes in batches using a real-time fluorescent quantitative PCR instrument with fluorescent melting curve analysis software. The base-quenching probe used does not require a quenching group, the synthesis of the tag sequence is relatively simple, the reaction reagent is easy to obtain, and the cost is low.

This study was mainly based on the standards of genetic screening for hereditary deafness published in the Chinese Medical Journal in 2021^50^, and the results have reference value for developing genetic testing projects for deafness in Changzhou City, Jiangsu Province. The established 2D-PCR detection technology is simple to operate and rapid, and the detection results at each site do not interfere with each other. Based on the differences in the frequencies of mutation sites in deafness-related genes in different regions, sites that need to be screened can be freely selected to reduce external costs. The limitation of this study is that we have established a 2D-PCR method for detecting nine hotspot mutations in the deafness susceptibility gene, which does not include the 35delG mutation. Considering that the average carrier frequency of the 35delG mutation is highest in Southern Europe and lowest in Eastern Asia^51^, and that the 35delG mutation is not common among the Chinese population^52^, the detection method developed in this study is particularly suitable for the Chinese population and not for Southern Europe. Our future goal is to improve and expand the detection range to cover fifteen key hotspot mutations, thereby facilitating genetic screening for deafness across different populations worldwide. In a follow-up study, more mutation sites of deafness-related genes will continue to be optimised, and more comprehensive screening sites will be established to identify the cause as soon as possible. This may aid in scientific prevention, help avoid inducements, delay deafness, and even aid in the selection of scientific treatment methods to fundamentally reduce birth defects.

### Supplementary Information

Supplementary Information 1.

Supplementary Information 2.

Supplementary Table 1.
