# Supplementary figures and images for: A novel method for detecting nine hotspot mutations of deafness genes in one tube
Source: Sci Rep. 2024 Jan 3;14:454. doi: 10.1038/s41598-023-50928-1 (PMC10764868; doi:10.1038/s41598-023-50928-1)

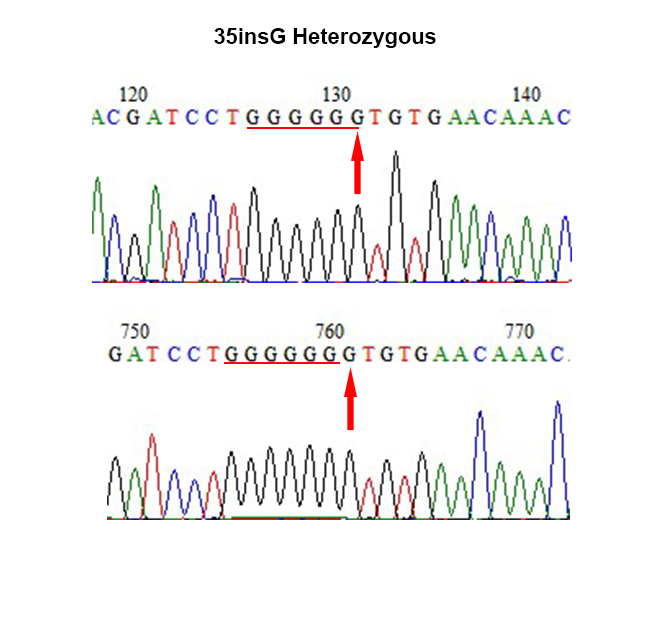

Supplement: Supplementary file 1 — Supplementary Information 1. [file 41598_2023_50928_MOESM1_ESM.jpg]
