# Supplementary material for: A novel method for detecting nine hotspot mutations of deafness genes in one tube
Source: Sci Rep. 2024 Jan 3;14:454. doi: 10.1038/s41598-023-50928-1 (PMC10764868; doi:10.1038/s41598-023-50928-1)
Supplement: Supplementary file 2 — Supplementary Information 2. [file 41598_2023_50928_MOESM2_ESM.docx]

Supplementary Fig 1. One patient with *GJB2* gene c.35insG was found by Sanger sequences. Red arrow points to the mutation site.
