# Supplementary material for: A novel method for detecting nine hotspot mutations of deafness genes in one tube
Source: Sci Rep. 2024 Jan 3;14:454. doi: 10.1038/s41598-023-50928-1 (PMC10764868; doi:10.1038/s41598-023-50928-1)
Supplement: Supplementary file 3 — Supplementary Table 1. [file 41598_2023_50928_MOESM3_ESM.docx]

**Supplementary Table 1. Volume of each primer in the reaction system**

| Primer Name | Forward primer (W)  Volume (µL) | Forward primer (M)  Volume (µL) | Reverse primer  Volume (µL) |
| --- | --- | --- | --- |
|  |  |  |  |
| c.919-2A>G | 0.15 | 0.15 | 1 |
| c.299-300delAT | 0.2 | 0.15 | 1 |
| c.176-191dell6 | 0.1 | 0.05 | 0.4 |
| c.235delC | 0.15 | 0.05 | 0.6 |
| c.1229C > T | 0.2 | 0.1 | 0.8 |
| c.2168A > G | 0.15 | 0.1 | 1 |
| c.538C >T | 0.05 | 0.2 | 1 |
| m.1494C > T | 0.05 | 0.1 | 0.6 |
| m.1555A > G | 0.05 | 0.05 | 0.3 |

W-Wild type; M-Mutant type
